# Tead1a Initiates Transcriptional Priming Through the TEAD1a/YAP‐Notch1‐Spi1/Cebpα Axis to Promote Neutrophil Fate

**DOI:** 10.1002/advs.202505441

**Published:** 2025-08-28

**Authors:** Wang Yiqin, Yang Ruimeng, Wang Peihong, Liu Xiaohui, Yuan Hao, Huang Hongli, Zhou Yongjian, Xie Baoshu

**Affiliations:** ^1^ Department of Gastroenterology and Hepatology Guangzhou First People's Hospital the Second Affiliated Hospital of South China University of Technology Guangzhou 510080 China; ^2^ Center for Medical Research on Innovation and Translation Guangzhou First People's Hospital School of Medicine South China University of Technology Guangzhou 510080 China; ^3^ CNRS‐LIA Hematology and Cancer Sino‐French Research Center for Life Sciences and Genomics State Key Laboratory of Medical Genomics Rui‐Jin Hospital Shanghai Jiao Tong University School of Medicine Shanghai 200000 China; ^4^ Department of Hematology Guangzhou First People's Hospital School of Medicine South China University of Technology Guangzhou 510080 China; ^5^ Department of Neurosurgery The First Affiliated Hospital of Sun Yat‐sen University Guangzhou 510080 China

**Keywords:** developmental hematopoiesis, neutropenia, single‐cell sequencing, transcriptional priming, TEAD1a

## Abstract

Clinical neutropenia, a blood disorder marked by faulty neutrophil production, resists effective treatment due to developmental bottlenecks in granulopoiesis. While the current therapy, such as granulocyte colony‐stimulating factor (G‐CSF), boosts neutrophil counts, its late‐stage action mobilizes dysfunctional cells, underscoring the need for early‐lineage therapeutic interventions. Leveraging zebrafish models, we found that transcriptional enhanced associate domain 1a (TEAD1a) initiates transcriptional priming to govern neutrophil lineage specification preceding hematopoietic stem cell formation. Genetic ablation of TEAD1a or disruption of its interaction with Yes‐associated protein 1 (YAP1) induces profound neutropenia. Mechanistic interrogation reveals that TEAD1a/YAP1 complexes potentiate Notch1‐mediated signaling to activate a Spi1/Cebpα transcriptional cascade during myeloid progenitor specification. This study uncovers a novel developmental regulatory window for myeloid lineage commitment and demonstrates the evolutionarily conserved role of TEAD1a‐mediated transcriptional priming in orchestrating neutrophil development. The discovery of this ultra‐early regulatory node provides a molecularly defined target for generating developmentally competent neutrophils, offering an innovative therapeutic strategy for refractory neutropenia.

## Graphical abstract

This study discovered that the protein TEAD1a is crucial for neutrophil development. In zebrafish models, disrupting TEAD1a or its interaction with partner protein YAP1 caused severe neutrophil deficiency. TEAD1a functions during the early HSC stage, activates *Notch1* signaling in the GMP stage, and triggers *Spi1* and *Cebpα* to drive neutrophil terminal differentiation and production. This identifies TEAD1a as a potential new target for treating neutropenia.

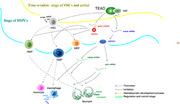

## 1 Introduction

Neutrophils constitute the first line of cellular immunity, yet clinical neutropenias pose considerable clinical risks and poor therapeutic outcomes.^[^ ^1^, ^2^, ^3^ ^]^ Current granulocyte colony‐stimulating factor (G‐CSF) therapies, while elevating neutrophil counts, often mobilize developmentally compromised cells through late‐stage differentiation pressure, underscoring the unmet need for early‐lineage intervention strategies.^[^ ^4^ ^]^

For the study of myeloid differentiation, the zebrafish model is an ideal choice because of its high degree of congruence with mammalian systems and its ability to perform large‐scale genetic and chemical screens to reveal the influences that regulate vertebrate hematopoiesis.^[^ ^5^ ^]^ Similar to humans, hematopoiesis in zebrafish is sequentially divided into two continuous and overlapping processes: primitive hematopoiesis and definitive hematopoiesis.^[^ ^6^, ^7^ ^]^ During primitive hematopoiesis, the lateral plate mesoderm (LPM) generates myeloid‐primed progenitors through transient transcriptional activation of lineage‐specific genes as early as 12 h post‐fertilization (hpf), preceding the emergence of definitive hematopoietic stem and progenitor cells (HSPCs) emergence through endothelial‐hematopoietic transition (EHT, ≈26 hpf).^[^ ^5^, ^8^ ^]^ Then, definitive HSPCs differentiate into various hematopoietic precursor cells.^[^ ^9^, ^10^, ^11^ ^]^ with a molecular network governing every stage that involves an interplay between multiple lineage‐specific transcription factors/cofactors and other epigenetic modifiers.^[^ ^12^, ^13^ ^]^ Crucially, transcriptional priming, an epigenetic or transcriptional pre‐patterning process that determines subsequent differentiation trajectories, has been established during or prior to the formation of hematopoietic stem cells (HSCs) or HSPCs.^[^ ^14^, ^15^ ^]^ In our study, zebrafish transcriptional enhanced associate domain 1a (TEAD1a) appears to initiate transcriptional priming early in embryonic development, thereby influencing HSPCs to significantly promote myeloid development and neutrophil fate.

The TEAD family modulates the most integral regulatory responses of the Hippo signaling pathway and exerts considerable influence over cellular growth, organ size regulation, and the progression of tumor malignancy.^[^ ^16^ ^]^ TEAD proteins form complexes with Yes1 associated transcriptional regulator/Transcriptional co‐activator with PDZ‐binding motif (YAP/TAZ) to form heterodimers that govern downstream gene expression. Four human TEAD proteins have been discovered: ranging from TEAD1 to TEAD4, and each features an N‐terminal TEA domain for a DNA binding domain and a C‐terminal domain responsible for transcriptional activation.^[^ ^17^ ^]^ These proteins regulate cellular growth, differentiation, and developmental processes during transcription. While the role of TEAD1 in cell growth regulation and tumor progression has been frequently documented, its impact on the hematopoietic system and the mechanisms involved remain understudied.

TEAD proteins exhibit elevated expression in the early stages of zebrafish embryogenesis during gastrulation, facilitating the differentiation and development of HSCs.^[^ ^18^ ^]^ However, current research on the role of TEAD in the differentiation and development of various other hematopoietic cells is quite limited, and further exploration is urgently needed.

In this study, we initially discovered that the high expression of *tead1a* can specifically stimulate neutrophil development, while knockdown and mutation of *tead1a* lead to specific neutrophil defects in zebrafish. However, *tead1a* is scarcely expressed in myeloid cells or neutrophils. Further research revealed that *tead1a* begins to function at an extremely early stage of hematopoietic development and sequentially promotes the differentiation and development of various cell stages through the TEAD/YAP‐Notch Homolog 1 (Notch1)‐ Spleen focus forming virus proviral integration oncogene 1 (Spi1 or pu.1)/ CCAAT/enhancer binding protein alpha (Cebpα) axis, ultimately driving neutrophil differentiation in zebrafish. TEAD1 activates via transcriptional priming early in embryogenesis, influencing HSPCs to promote myeloid development and neutrophil fate, and presenting an emerging target for neutrophil deficiency therapy.

## 2 Results

### 2.1 Overexpression of *Tead1a* Caused an Increase in HSPCs and Neutrophils in Zebrafish

During zebrafish embryogenesis, TEAD proteins show elevated expression levels during the gastrulation stage and near the EHT stage.^[^ ^18^ ^]^ Furthermore, the down‐regulated TEAD inhibited the production of hematopoietic progenitors when it happened in embryoid bodies in vitro or the aorta‐gonad‐mesonephros region (AGM)‐derived *fetal liver kinase 1*‐positive (*flk1* ^+^) endothelium cultured ex vivo.^[^ ^18^ ^]^ Based on these reports, we began to study whether TEAD1 had the ability to initiate the transcriptional priming of HSPCs differentiation. Briefly speaking, the RNA fragment of zebrafish *tead1a* was injected into zebrafish embryos at the one‐cell stage. Whole‐mount mRNA in situ hybridization (WISH) analysis was conducted with a series of lineage‐specific markers in early‐stage embryos to evaluate the effects of highly expressed *tead1a* on hematopoietic differentiation and lineage commitment.

After the primitive hematopoietic precursors were generated from the LPM in the yolk sac of the zebrafish embryo, some blood progenitors and vascular progenitors emerged from the intermediate cell mass (ICM) and reached their proliferation peak at 20–26 hpf. The vascular progenitors formed two primary axial vessels: the dorsal aorta (DA) and the posterior cardinal vein. The blood progenitors differentiate into primitive blood cells to fill these new vessels.^[^ ^5^ ^]^

**Figure 1:**
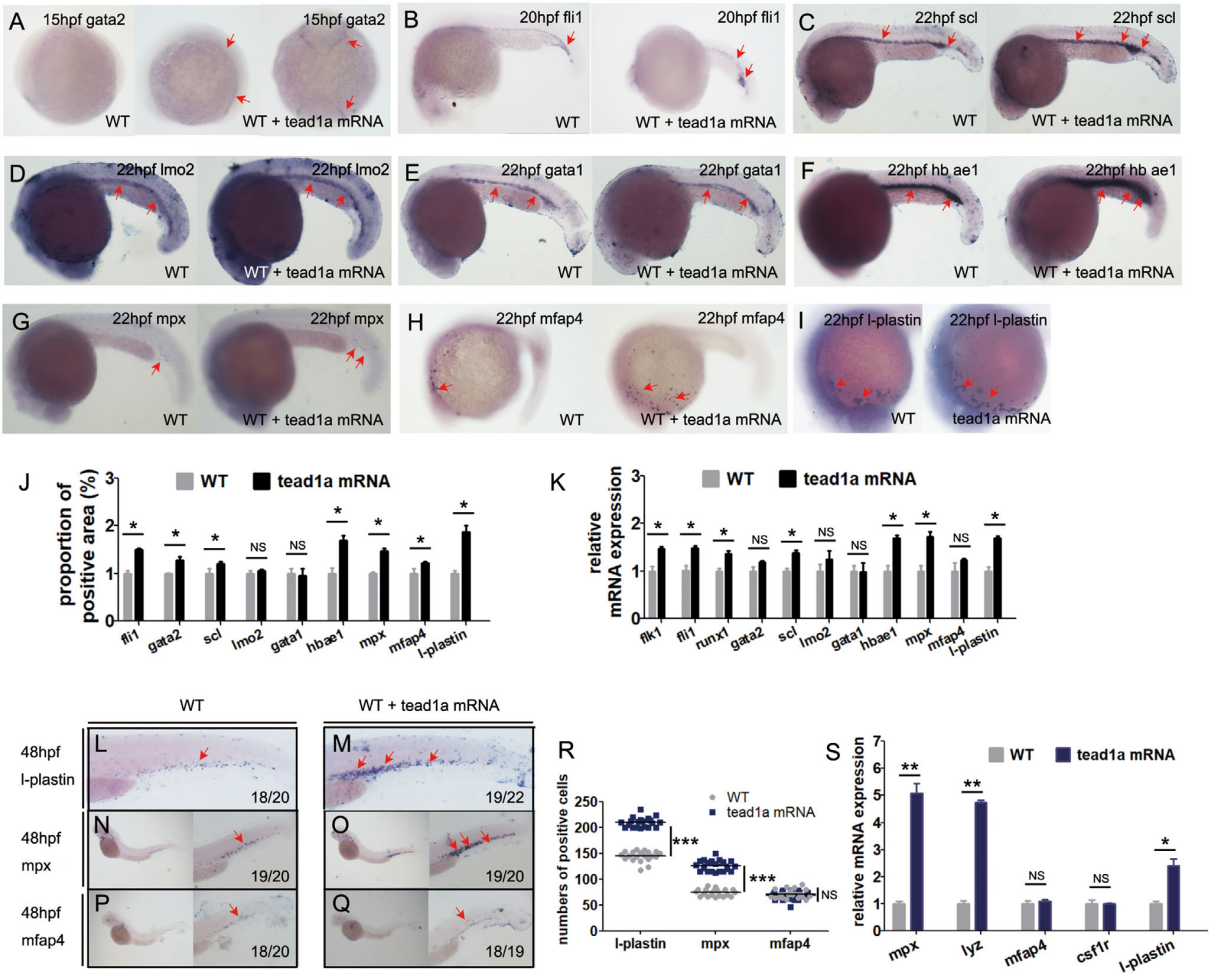
Over‐expression of *tead1a* caused an increase in many HSPCs in zebrafish. A–J) Whole‐mount in situ hybridization (WISH) analysis of vascular endothelial cell marker *fli1* (B), stem cell lineage markers *gata2* (A) and *scl* (C); erythroid lineage markers *lmo2* (D), *gata1* (E) and hemoglobin marker *hbae1* (F); myeloid lineage markers *mpx* (G), *mfap4* (H) and *l‐plastin* (I), and the statistical results (J). K) QPCR analysis of 15–22 hpf zebrafish embryos for HSPC markers. (L‐R) WISH analysis of neutrophil marker *mpx* L,M), monocyte/macrophage marker *mfap4* N,O), and myelocyte marker *l‐plastin* P,Q) at 48 hpf, and the statistical result (R). (S) QPCR analysis of 48 hpf zebrafish larvae for these markers. Error bars represent the mean ± standard deviation (SD) of at least 15–40 embryos or larvae from three independent experiments. Statistical significance was calculated using Student's *t*‐test. *, *p* <0.05; **, *p* <0.01; ***, *p* < 0.001; NS, not significant.

In our study, after *tead1a* was mimicly highly expressed, the *gata2^+^* hematopoietic precursors were first promoted in the LPM at 15 hpf, then the *fli1^+^* vascular endothelial cells increased in the ICM at 20hpf, and the *stem cell leukemia transcription factor*‐positive (*scl* ^+^ or *tal* ^+^) HSCs increased at 22 hpf (**Figure** **1**A–C,J). As to the primitive hematopoietic precursors, the *GATA binding protein 1*‐positive (*gata1* ^+^) and *LIM domain only 2*‐positive (*lmo2* ^+^) erythroid lineages didn't change significantly, though the hemoglobin gene was upregulated (Figure 1D–F,J). The *lymphocyte cytosolic protein 1*‐positive (*l‐plastin* ^+^)  myeloid lineage and the *myeloperoxidase*‐positive (*mpx* ^+^ or *mpo* ^+^) granulocytic lineage increased largely, while the *microfibrillar‐associated protein 4*‐positive (*mfap4* ^+^) macrophage lineage did not change significantly (Figure 1G–I,J). A consistent result was found when the zebrafish were taken embryos at 22 hpf for whole‐embryo quantitative polymerase chain reaction (qPCR)enhanced green fluorescent protein (Figure 1K).

Subsequently, HSPCs began to be produced exclusively from EHT at 26 hpf^[^ ^8^ ^]^ and then emerged in the aorta‐gonad‐mesonephros region (AGM region) from 48 hpf to 7 days post‐fertilization (dpf), and appeared in the caudal hematopoietic tissue (CHT) or posterior blood island (PBI),^[^ ^19^ ^]^ where the HSPCs further differentiated into various hematopoietic lineages.^[^ ^20^, ^21^ ^]^

Overexpression of *tead1a* in our research significantly enhanced neutrophil development in the CHT during definitive hematopoiesis. While *l‐plastin* ^+^ pan‐myeloid cells (including both neutrophils and macrophages) increased markedly in *tead1a*‐overexpressing larvae, this expansion was specifically attributed to *mpx* ^+^ neutrophils, with *mfap4* ^+^ macrophage numbers remaining stable (Figure 1L–R). These findings were corroborated by qPCR analysis of 2 dpf zebrafish larvae (Figure S1, Supporting Information).

Briefly speaking, *tead1a* overexpression drives HSPCs expansion and specifically enhances neutrophil production during definitive hematopoiesis.

### 2.2 *Tead1a* Knockdown Impacted Erythroid Lineage, Disrupted Myeloid Lineage, and Related Inflammatory Responses to Injury in Zebrafish

First, *tead1a* morpholino (MO) was injected into zebrafish embryos to establish the tead1a knockdown phenotype.^[^ ^22^, ^23^ ^]^ WISH analysis revealed transient upregulation of erythropoiesis in *tead1a* morphants, though *gata1* expression showed no significant difference from wild‐type (WT) at 22 hpf. At 48 hpf, *hbae3 globin* expression showed modest upregulation by WISH, corroborated by increased hemoglobin levels through O‐dianisidine staining (Figure S1A–C, Supporting Information).

**Figure 2:**
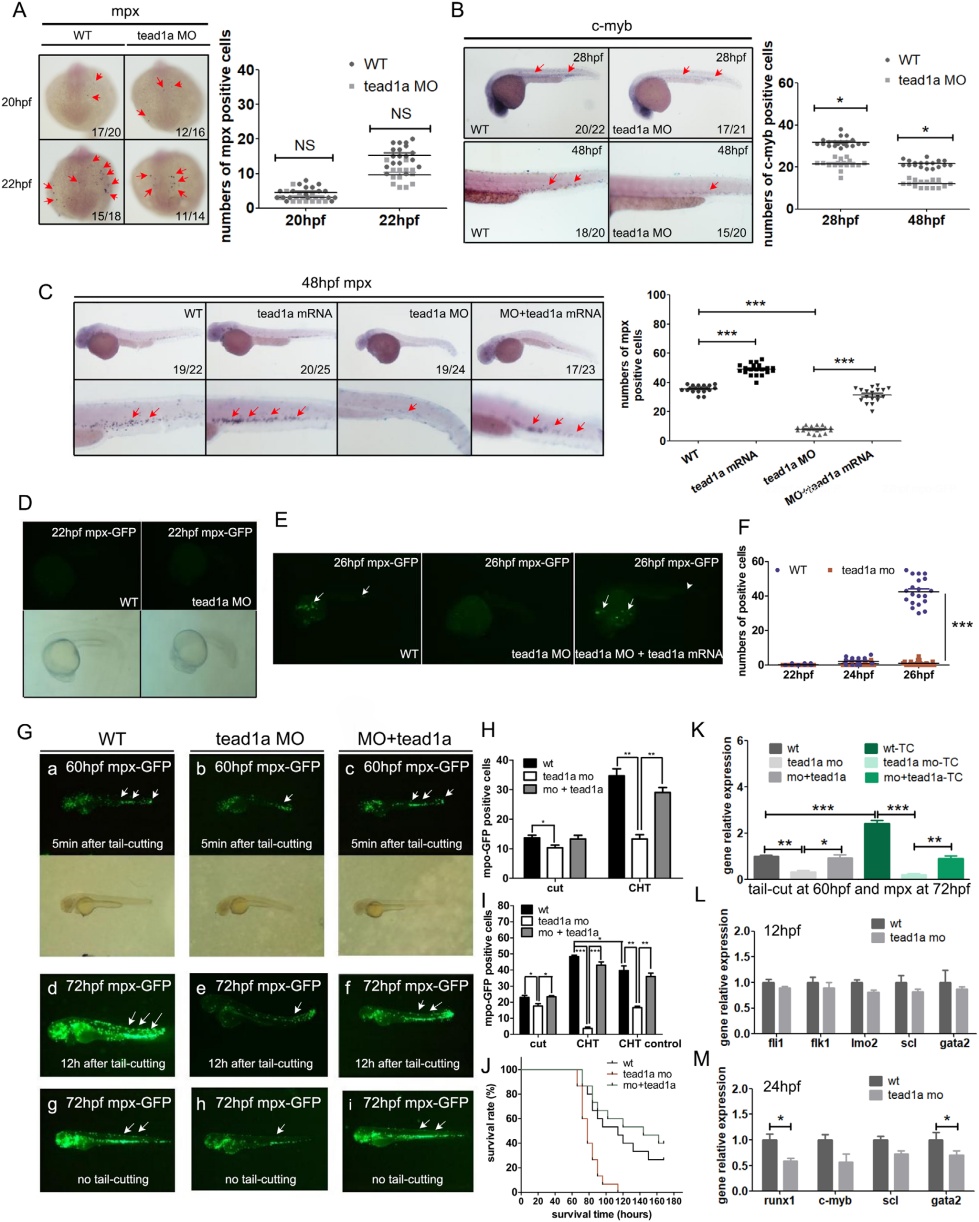
*Tead1a* knockdown disrupts neutrophils in zebrafish both quantitatively and functionally. A) WISH analysis shows that *tead1a* knockdown leads to a decrease of *mpx*‐positive primitive neutrophils at ≈20–22 hpf. B) WISH analysis shows that *tead1a* knockdown leads to a reduction of *c‐myb*‐positive stem cells from 28 hpf to 48 hpf. C) At 48 hpf, *mpx*‐positive maturer neutrophils were significantly increased in *tead1a*‐overexpress embryos, decreased in *tead1a*‐knockdown embryos, and the despaired phenotype can be rescued by *tead1a* mRNA sequence micro injection in WISH analysis. D–F) *Tead1a* MO begins to be detected decreasing neutrophil quantity at ≈24–26 hpf in the line of Tg(*mpx*:eGFP). G–K) *Tead1a* knockdown disrupts neutrophils in zebrafish quantitatively and functionally in the line of Tg(*mpx*:eGFP): (G) the detection by fluorescence microscope; (H, I) the sub‐group statistical results; (J) the survival results; (K) the qPCR results. L) At 12 hpf, the expression changes of *fli1*, *flk1*, *lmo2*, *scl*, and *gata2* in *tead1a* MO group were determined using qPCR. M) At 24 hpf, the expression changes of *runx1, c‐myb*, *scl*, and *gata2* in *tead1a* MO group were determined using qPCR. The CHT control (I) means the neutrophils in CHT region without tail‐cut as control (G g‐i). TC (K) means tail‐cut. Error bars represent the mean ± standard deviation (SD) of at least 15–40 embryos or larvae from three independent experiments. Statistical significance was calculated using the Student's *t*‐test. *, *p* <0.05; **, *p* <0.01; ***, *p* < 0.001; NS, not significant.

Furthermore, WISH analysis suggested a marginal reduction of *mpx^+^* primitive neutrophils in the morphants at 20–22 hpf, though not statistically significant (**Figure** **2**A). A progressive decline in the hematopoietic stem cell marker cellular myeloblastosis viral oncogene homolog (*c‐myb*) was observed in morphants from 28 to 48 hpf (Figure 2B). At 48 hpf, the *tead1a*‐overexpressing embryos exhibited significantly increased *mpx* ^+^ mature neutrophils, while morphants showed reduced numbers, with both phenotypes being rescued by *tead1a* mRNA sequence co‐injection (Figure 2C).

For better detection, we utilized *tead1a* MO and/or *tead1a* mRNA sequence in the line of Tg(*mpx*:enhanced green fluorescent protein, eGFP).^[^ ^24^, ^25^ ^]^ In this line, *mpx*‐eGFP positive neutrophils were not visible until 24–26 hpf; *tead1a* MO led to a decrease in neutrophils, which could be rescued by *tead1a* mRNA sequence injection (Figure 2D–F).

The tail‐cut model reflects damage repair functions under biological stress conditions.^[^ ^26^, ^27^ ^]^ In the neutrophil fluorescently labeled transgenic fish line Tg(*mpx*: eGFP), 5 minutes after tail‐cut at 60 hpf, neutrophils accumulated more at the wound site in the WT group than in the *tead1a* MO group, and the WT group had a seemingly higher proportion of neutrophils in CHT than the MO group (Figure 2G a‐c,H). Twelve hours after tail‐cut, the number of neutrophils in the CHT area of WT zebrafish larvae increased significantly, while there was hardly any change in the CHT of the *tead1a* MO group. Compared with WT, the zebrafish in the MO group exhibited significantly defective systemic neutrophil‐associated inflammatory response and subsequent compensatory neutrophil differentiation in the CHT region (Figure 2G d‐i,H,I). The *tead1a* MO group had a worse prognosis and could be rescued by *tead1a* mRNA injection (Figure 2J).

This difference was not limited to the fluorescent transgenic fish lines and the WISH analysis we visually observed. We collected normal zebrafish embryos at several time points of primary and definitive hematopoiesis, as well as their 60 hpf tail‐cut models, for whole‐embryo qPCR analysis and obtained consistent results of *mpx* expression (Figure 2K). Furthermore, at the 72 hpf stage, 12 h after tail‐cut, compared to the control group, the expression of granulocyte markers *cebpα* and *lyz* in the MO group zebrafish larvae significantly decreased, and the expression of neutrophil‐related inflammatory cytokines gene *il‐1β* and *il‐6* also significantly decreased. Neither of these markers above could be increased by tail‐cut injury stimulation, and this abnormal expression could be rescued by *tead1a* mRNA injection. The macrophage markers *mfap4* and *macrophage expressed gene (mpeg1)* expression showed no difference between the control and MO groups, and both could be up‐regulated by tail‐cut injury stimulation (Figure S1D–F, Supporting Information). In another whole‐embryo qPCR analysis of HSCs and HSPCs markers, at the 12 hpf stage, there was a slight decrease in the expression of endothelial cell or HE markers *fli1* and *flk1* in the MO group zebrafish embryos and a slight reduction in the expression of hematopoietic stem cell markers *lmo2*, *scl*, and *gata2*. Still, none of these differences were statistically significant compared to the WT group (Figure 2L). At the 24 hpf stage, there was a more seeable decrease in the expression of hematopoietic stem cell markers *runx1*, *c‐myb*, *scl*, and *gata2*, among which the *runx1* and *gata2* were downregulated significantly compared to the WT group (Figure 2M). These results all implied the mild down‐regulation of HSCs, significant defective neutrophil differentiation, and related destroyed inflammatory response in *tead1a* down‐regulation zebrafish.

### 2.3 Construction of Zebrafish Line with *Tead1a* Mutation

**Figure 3:**
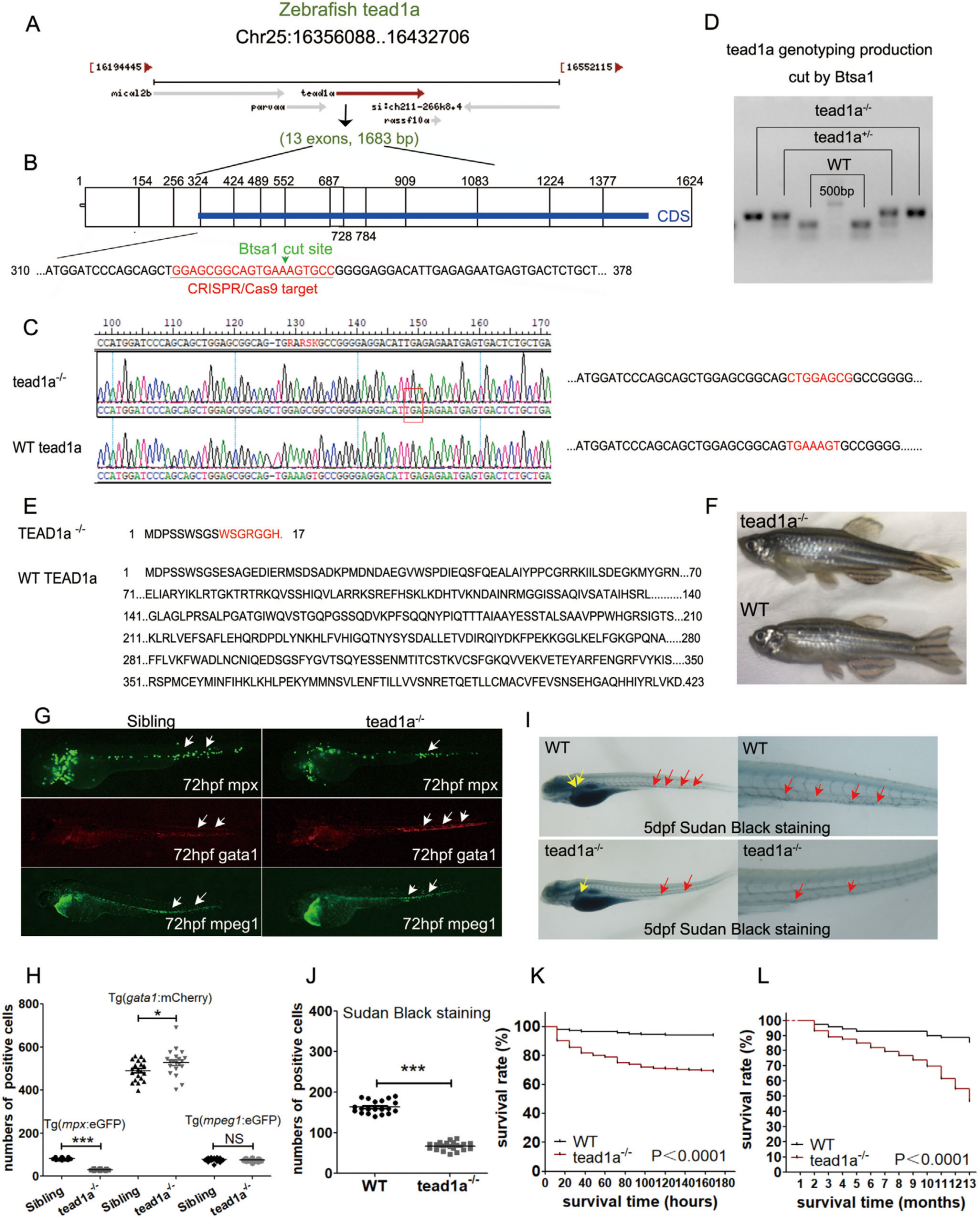
Construction of the *tead1a*‐mutant zebrafish line and gross analysis of its phenotype. (A‐F) Construction of the *tead1a* ‐mutant zebrafish line. A,B) Location (A) and structure (B) of the zebrafish *tead1a* gene in WT and mutant model, the CRISPR/Cas9 target sequence is highlighted in red letters and the restriction enzyme Bts1 restriction site is shown by a green arrow. C) Sanger sequencing reveals differences in the *tead1a* gene sequences between the WT and mutant zebrafish lines, confirming successful construction. D) Genotyping of the *tead1a* ‐mutant zebrafish line by the cut of Bts1. (E) Different amino acid sequences between the WT and mutant zebrafish lines. F) Offspring of the *tead1a* mutant stable, genetically inherited adult zebrafish, which was gained by crossing with WT, followed by selection and subsequent self‐crossing. G,H) Construction and analysis of the lines *tead1a^‐/‐^*//Tg(*mpx*:eGFP), *tead1a^‐/‐^* //Tg(*gata1*:DsRed), and *tead1a^‐/‐^* //Tg(*mpeg1*:eGFP). I,J) Sudan Black staining of *tead1a* mutants at 5 dpf. Error bars represent the mean ± standard deviation (SD) of at least 15–40 embryos or larvae from three independent experiments. Statistical significance was calculated using the Student's *t*‐test. *, *p* <0.05; **, *p* <0.01; ***, *p* < 0.001; NS, not significant.

To explore epigenetic regulation in zebrafish from embryogenesis to adulthood, we conducted systematic clustered regularly interspaced short palindromic repeats (CRISPR)/ CRISPR‐associated protein 9 (Cas9) (CRISPR/Cas9)‐mediated genome editing of epigenetic genes on zebrafish chromosomes.^[^ ^28^ ^]^ We designed single‐guide RNA (sgRNA) to target the first exon of *tead1a* (**Figure** **3**A,B). An 7‐nucleotide sequence “TGAAAGT” was transformed into “CTGGAGCG” in *tead1a^−/−^* mutants due to a insertion mutation (Figure 3C). This mutation prevented digestion by the restriction enzyme Bts1s, used for genotyping (Figure 3D), and was predicted to introduce a frameshift mutation from amino acid 9, leaving almost no functional domain, including the TEF domain (Figure 3E). The appearance of adult *tead1a^−/−^* mutants exhibited a slimmer and sicker phenotype compared to WT individuals (Figure 3F, general observation).

We crossed *tead1a^−/−^* mutants with the lines Tg(*mpx*:eGFP), Tg(*gata*1:DsRed), and Tg(*mpeg1*:eGFP), respectively, and self‐crossed them, respectively, to select offspring that stably express, namely build up the lines *tead1a^−/−^*//Tg(*mpx*:eGFP), *tead1a^−/−^*//Tg(*gata1*:DsRed), and *tead1a^−/−^*//Tg(*mpeg1*:eGFP). In these lines, eGFP^+^ neutrophils decreased at 72 hpf, while DsRed^+^ erythrocytes and eGFP^+^ macrophages remained unchanged, confirming that the *tead1a* mutation specifically impaired neutrophil hematopoietic development (Figure 3G,H). Sudan Black staining^[^ ^29^ ^]^ at 5 dpf confirmed the reduced neutrophil population in the CHT and kidney marrow of the *tead1a* mutants (Figure 3I,J). The *tead1a^−/−^* mutants had survival defects before 24 hpf and after six months post‐fertilization compared to WT individuals (Figure 3K,L).

Flow cytometry quantified neutrophils in 72 hpf zebrafish larvae using the line of Tg(*mpx*:eGFP). The results showed a 71.4% reduction in eGFP‐positive cells in *tead1a^−/−^*//Tg(*mpx*:eGFP) larvae at 72 hpf compared to controls (Figure S2, Supporting Information). However, the low neutrophil count prevented accurate whole‐transcriptome sequencing after sorting.

### 2.4 Zebrafish *Tead1a* Mutation Led to Increased Erythrocytes and Significantly Decreased Neutrophils

HSPCs differentiate into various hematopoietic lineages, gradually migrate to the thymus ≈54 hpf, and to the anterior region of kidney marrow ≈4 dpf, which serves as bone marrow in mammals.^[^ ^30^ ^]^

**Figure 4:**
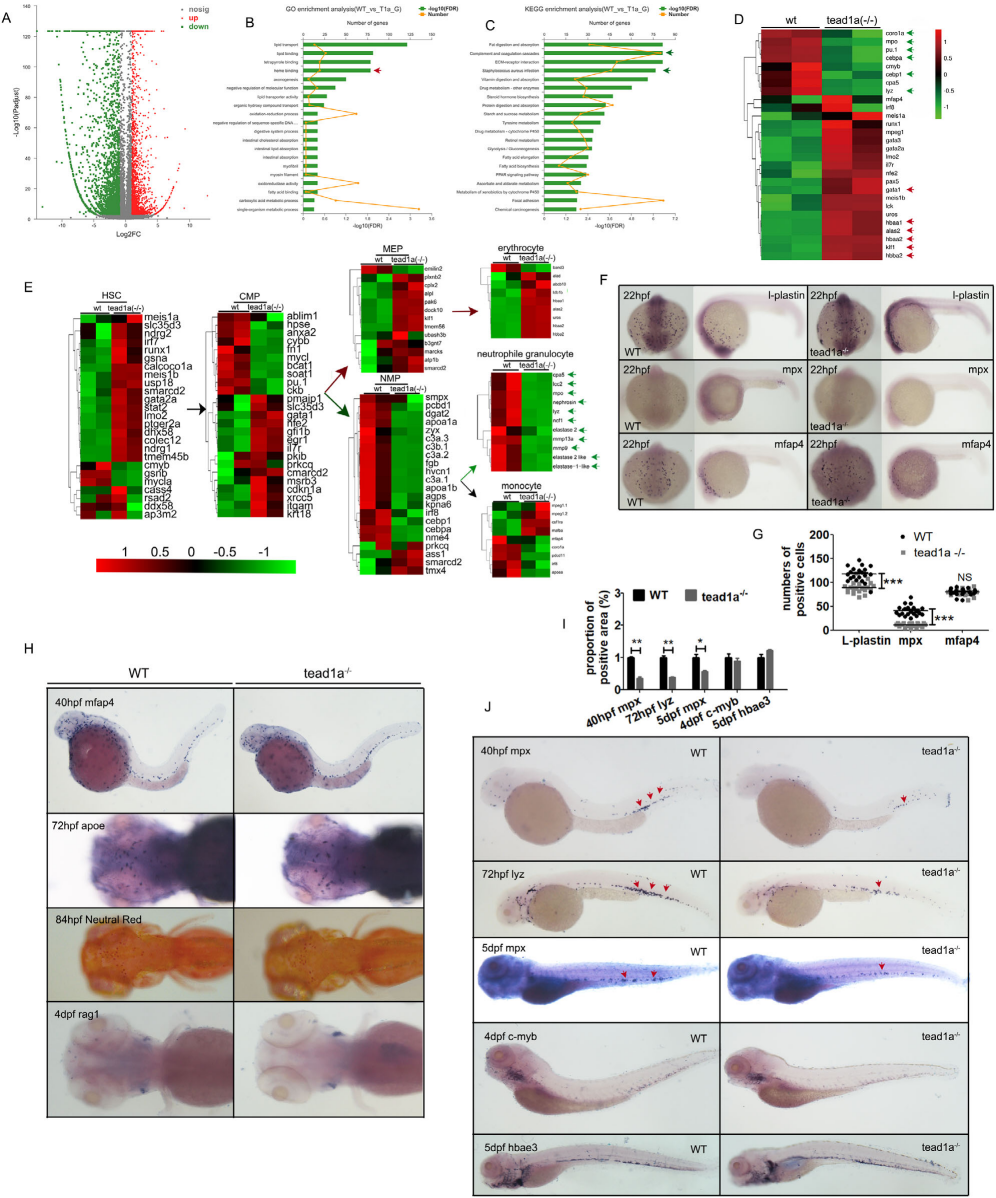
Gross analysis of *tead1a*‐mutant (*tead1a* ^‐/‐^) zebrafish in hematopoiesis. A–E) Whole transcriptome sequencing of the adult *tead1a* ^‐/‐^ zebrafish kidney marrow; (A) The volcano plot of differentially expressed genes, (B‐C) GO and KEGG analyses of the enriched genes. (D‐E) Further results of RNA‐sequencing of kidney marrow, largely differently expressed genes presented by heat maps. F,G) WISH analysis verified the neutropenia profile in *tead1a* ^‐/‐^ zebrafish embryos at 22 hpf. H) WISH and Neutral Red staining analysis of macrophages, microglial cells, various phagocytes, and lymphoid cells in parotid *tead1a* ^‐/‐^ zebrafish larvae. I,J) WISH analysis of neutrophils, HPSCs and erythrocytes. Error bars represent the mean ± standard deviation (SD) of at least 15–40 embryos or larvae from three independent experiments. Statistical significance was calculated using the Student's *t*‐test. *, *p* <0.05; **, *p* <0.01; ***, *p* < 0.001; NS, not significant.

We performed whole transcriptome sequencing using the anterior region of kidney marrow from the adult *tead1a* ^‐/‐^ line, resulting in a volcano plot of differentially expressed genes (**Figure** **4**A). Subsequent Gene Ontology (GO) and Kyoto Encyclopedia of Genes and Genomes (KEGG) analysis of the enriched genes revealed significant enrichment in pathways associated with heme synthesis, complement activation, and signaling related to bacterial infection (Figure 4B,C), which was related to our results about myeloid and erythroid lineage. In further hematopoietic results of RNA‐sequencing of kidney marrow, markers of neutrophils were largely down‐regulated, such as *coro1a*, *mpx/mpo*, *spi1/pu.1*, *cebpα*, *cebp1*, and *lyz/lysozyme C* in the *tead1a* mutants, which implied the neutropenia of bone marrow origin (Figure 4D, green arrow). Other genes with reduced expression were *cpa5* and *c‐myb*. Although a marker of mast cell lineage, the *carboxypeptidase a5 (cpa5)* gene is dependent on Notch signaling and can co‐localize with *spi1*, *mpx*, *l‐plastin*, and *lyz* rather than *cebpα* in zebrafish,^[^ ^31^, ^32^, ^33^ ^]^ which suggests a distinct myeloid population differentiates from a common granulocyte/monocyte progenitor in Notch signaling.^[^ ^34^, ^35^ ^]^ The abnormal activation of the *c‐myb* gene can lead to the expansion of abnormal granulocytes in zebrafish, which is similar to human myelodysplastic syndrome (MDS), implying that *c‐myb* is a pro‐myeloid HSPC marker.^[^ ^17^ ^]^ Markers of erythroid lineage were mildly up‐regulated, such as *gata1*, *5‐aminolevulinate synthase 2 (alas2)*, and several genes encoding different kinds of globin^[^ ^36^, ^37^ ^]^ (Figure 4D, red arrow).

Upon further analysis of the genes related to hematopoiesis, we generated a heatmap depicting the differential expression of key genes in the hematopoietic process. The mutation of *tead1a* led to alterations in the expression of most genes crucial for HSPCs. This mutation resulted in mile elevated expression of key erythroid lineage‐related hematopoietic genes and a pronounced reduction in the expression of genes essential for neutrophil development (Figure 4E; Data S1, Supporting Information “RNAseq of kidney marrow”).

WISH analysis verified the neutropenia profile in *tead1a* mutant (*tead1a* ^‐/‐^) zebrafish embryos at 22 hpf (Figure 4F,G). WISH or staining analysis recognized no significant differences in *mafap4* ^+^ macrophages, *apoe* ^+^ microglial cells, Neutral Red positive phagocytes of the nervous system, or *rag1* ^+^ lymphoid cells in parotid glands^[^ ^38^, ^39^, ^40^ ^]^ (Figure 4H). We found largely down‐regulated *mpx* and *lyz* in *tead1a* ^‐/‐^ zebrafish larvae in the whole body, particularly in CHT, verifying neutropenia syndrome (Figure 4I,J, red arrow). We also detected mildly decreased *c‐myb* ^+^ HSPCs and increased *hbae3* ^+^ erythrocytes in *tead1a* ^‐/‐^ larvae (Figure 4I,J), correlated with the RNA‐sequencing results in adult zebrafish kidney marrow.

### 2.5 The Mild Restraining Effect of TEAD1a on Erythropoiesis is Dependent on the TEAD/YAP Combination and *Gata1*, and Thus to Improve Non‐Erythroid Myeloid Hematopoiesis

We did not find significantly increased *hbae3* ^+^ erythrocytes in *tead1a* ^‐/‐^ larvae at 5 dpf, this could be due to the fact that we were not within the optimal observation window. The phenotype is more challenging to observe at 4–5 dpf compared to 3 dpf, so potentially we missed the details due to technical reasons. We deepened the models and continued researching.

**Figure 5:**
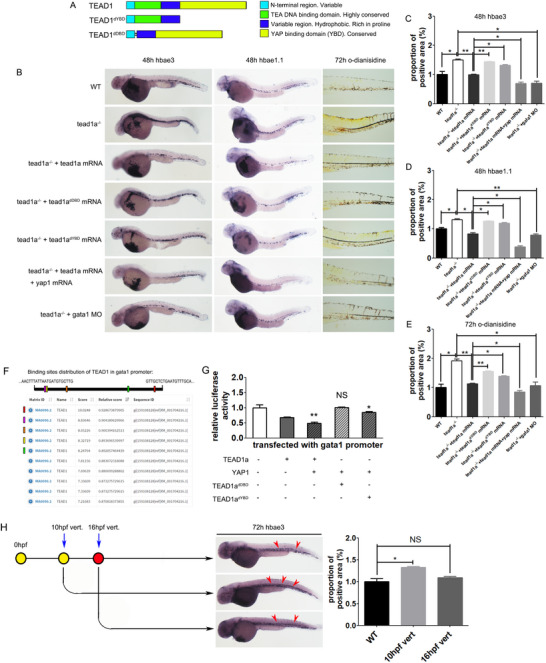
The mild restraining effect of TEAD1a on erythropoiesis. A) Structures of TEAD1a and its mutants. B–E) Assessment of TEAD1a's effect on erythropoiesis by WISH analysis and O‐dianisidine staining. F,G) Luciferase assay found that TEAD1a is capable of directly binding to the promoter sequence of *gata1* and suppressing its activity, this effect can be activated by the binding of YAP1. H) Inhibits TEAD‐YAP combining using verteporfin can mimic the effect of TEAD1a mutation, while it did not work after 10–16 hpf. Ver. For verteporfin. Error bars represent the mean ± standard deviation (SD) of at least 15–40 embryos or larvae from three independent experiments. Statistical significance was calculated using the Student's *t*‐test. *, *p* <0.05; **, *p* <0.01; ***, *p* < 0.001; NS, not significant.

TEAD1a included two crucial domains, featuring an N‐terminal TEA domain for DNA binding (DNA‐binding domain, DBD) and a C‐terminal domain tasked with transcriptional activation.^[^ ^17^ ^]^ YAP1 was widely reported to bind TEAD1 at a domain that highly overlapped with the C‐terminal domain, thus, it was also called the YAP‐binding domain (YBD).^[^ ^22^, ^41^, ^42^ ^]^ We first created TEAD1a mutants; we constructed a sequence devoid of the transcriptional activation domain encoding TEAD1a^dYBD^, and another sequence devoid of the DNA binding domain encoding TEAD1a^dDBD^ (**Figure** **5**A).

Then we began to detect the mutants’ rescue effects in the pro‐erythroid phenotypes in the tead1a^‐/‐^ line. When expanding the erythroid lineage markers and time windows, we detected an upregulated expression of *hbae3*, the gene encoding globin. The activated erythroid phenotype induced by the *tead1a* mutation can be rescued by the microinjection of *tead1a* mRNA. Still, it cannot be rescued by using a *tead1a* variant sequence that disrupts the DNA‐binding domain (*tead1a^dDBD^*) or a *tead1a* variant sequence (*tead1a^dYBD^*) that disrupts the transcriptional activation domain. This activated erythroid phenotype can be rescued by using *gata1* MO to mimic the low expression of *gata1*. Furthermore, *yap1* enhanced the repressive effect of *tead1a* on *hbae3* expression (Figure 5B,C). The same results were detected in the WISH analysis of *hbae1.1* expression and O‐dianisidine staining of the heme (Figure 5B,D,E).

After that, we constructed the promoter sequence of *gata1*, which contained multiple TEF binding sites predicted by bioinformatics software (Figure 5F). Upon co‐transfecting these constructs into 293T cells and conducting a luciferase assay, we discovered that TEAD1a is capable of directly binding to the promoter sequence of *gata1* and suppressing its activity. YAP1 was found to enhance the repressive effect of TEAD1a on the *gata1* promoter, and the effect was dependent on YBD. This may explain why TEAD1a^dYBD^ despaired the synergistic impact of TEAD1a and YAP1, and TEAD1a^dYBD^ despaired the basal depressive effect of TEAD1a on *gata1* expression (Figure 5G).

Furthermore, the mild erythroid‐promoting phenotype of the *tead1a* mutation can be mimicked by verteporfin, which specifically inhibits TEAD‐YAP complex formation to disrupt their downstream signals.^[^ ^18^ ^]^ However, this effect of verteporfin is time‐dependent: treating verteporfin at 10 hpf can activate erythropoiesis but not at 16 hpf (Figure 5H).

In summary, the biphasic regulation of TEAD1a in erythropoiesis, characterized by an initial activation followed by an inhibition phase, is associated with its role in the early stimulation of HSCs and the subsequent direct suppression of *gata1* expression. The inhibitory action of TEAD1a depends on binding directly to the promoter of *gata1* and is potentiated by YAP. TEAD1a down‐regulated *gata1* expression so as to improve the non‐erythroid myeloid hematopoiesis suppressed by *gata1*, mainly neutrophil and macrophage differentiations.

### 2.6 The Neutrophil‐Promoting Effect of TEAD is Dependent on the TEAD/YAP Complex Formation and the Notch Signaling Pathway

**Figure 6:**
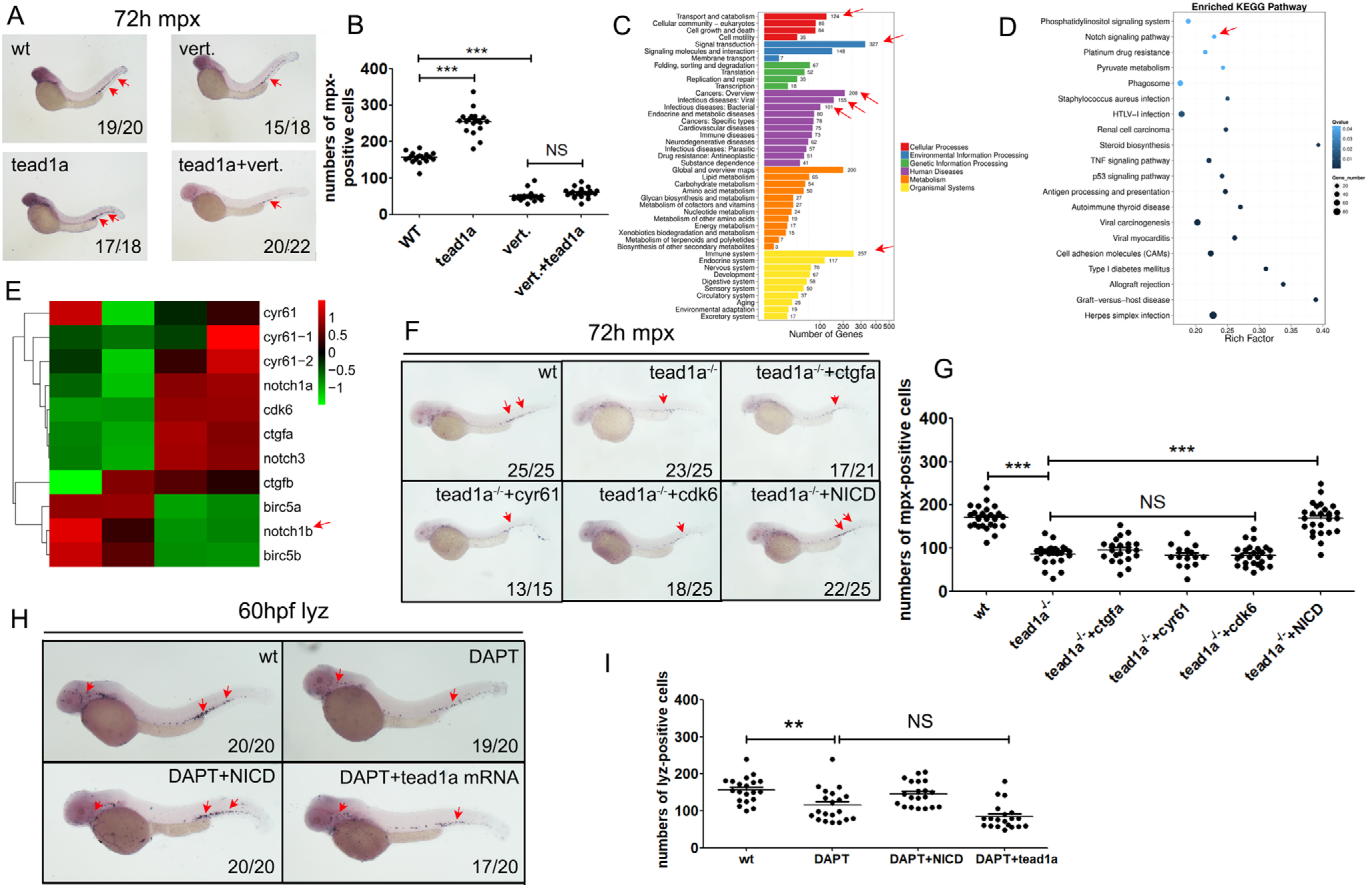
The neutrophil‐promoting effect of TEAD is dependent on the TEAD/YAP complex formation and the Notch signaling pathway. A,B) Verteporfin despaired the neutrophil development in zebrafish. C–E) Analysis of the 32D cl3 cells treated with verteporfin. (C‐D) The KEGG enrichment results of the whole transcriptome sequencing of the treated 32Dcl3 cells indicate the downregulation of multiple pathways, including the Notch signaling pathway. (E) The organization of the expression changes of key downstream genes of TEAD and genes of the Notch signaling pathway using a heatmap. F,G) NICD rescued the neutrophil defect phenotype following *tead1a* mutation. H,I) *Tead1a*‐overexpression could hardly rescue the neutrophil defect phenotype by DAPT. Ver. For verteporfin. Error bars represent the mean ± standard deviation (SD) of at least 15–40 embryos or larvae from three independent experiments. Statistical significance was calculated using the Student's *t*‐test. *, *p* <0.05; **, *p* <0.01; ***, *p* < 0.001; NS, not significant.

TEADs can be activated by YAP/TAZ compounds and then induce the expression of several Hippo pathway target genes, including*connective tissue growth factor* (*ctgf*), *cysteine‐rich angiogenic inducer* *61* (*cyr61*), and *axl* *receptor tyrosine kinase* (*axl*).^[^ ^43^, ^44^ ^]^ Verteporfin, a small molecule photosensitizer, effectively inhibits TEAD‐YAP combining by up‐regulating the YAP chaperon protein 14‐3‐3σ, thus sequestering YAP in the cytoplasm and targeting it for degradation.^[^ ^45^ ^]^ First, we verified whether verteporfin can despair the promotional effect of TEAD on neutrophil development. We examined the expression levels of *mpx* and/or *lyz* by WISH analysis and whole‐embryo qPCR after the micro‐injection of *tead1* and *yap* and treated with verteporfin. Consistent with our hypothesis, verteporfin similarly resulted in severe neutrophil defects in zebrafish embryos, which could hardly be rescued by overexpression of *tead1a* mRNA injection (**Figure** **6**A,B).

Because tead1a had a low expression in the qPCR results of both whole‐embryo and sorted neutrophils, we hypothesize that the role of TEAD1a in zebrafish neutrophils is part of a cascade amplification process. The initial effector molecules in this process likely originate from myeloid progenitor cells rather than from mature neutrophils. Based on this, we used murine myeloid progenitor 32D cl3 cells treated with verteporfin to clarify the effects of the tead1a or TEAD1/YAP complex on myeloid cells, especially which downstream signaling pathway is affected. In the GO enrichment results of the whole transcriptome sequencing of the verteporfin‐treated 32Dcl3 cells, the differentially expressed genes are mainly enriched in the “transport and catabolism,” “cancers: overview,” “infectious diseases: viral,” “infectious diseases: bacterial”, and “immune system” (Figure 6C). The KEGG enrichment results of the whole transcriptome sequencing of the treated 32Dcl3 cells indicated the downregulation of multiple pathways, including the Notch signaling pathway (Figure 6D; Data S2, Supporting Information “RNAseq of 32D cells”). We further organized the expression changes of key downstream genes of TEAD and genes of the Notch signaling pathway into a heatmap and found that *notch1b* was significantly downregulated (Figure 6E). Consistent with these results, the nuclear active intracellular domain of notch1b, the notch intracellular domain (NICD), can rescue the neutrophil defect phenotype following tead1a mutation (Figure 6F,G), while the neutrophil defect phenotype by *N‐[N‐(3,5‐Difluorophenacetyl)‐L‐alanyl]‐S‐phenylglycine t‐butyl ester* (DAPT, Sigma‐Aldrich) could hardly be rescued by *tead1a* mRNA sequence injection (Figure 6H,I). DAPT was used here to block the cleavage of Notch proteins to prohibit intracellular and nuclear translocation of the NICD.^[^ ^46^, ^47^ ^]^

### 2.7 The Influence of TEAD1a on Neutrophils Depends on TEAD/YAP‐Notch1‐Spi1/Cebpα Signaling and Phase, with no Significant Effect After 14–16 hpf. TEAD Promotes the Formation of Neutrophil Populations Before the Emergence of HSCs and Ensures Their Subsequent Proliferation, Differentiation, and Function

**Figure 7:**
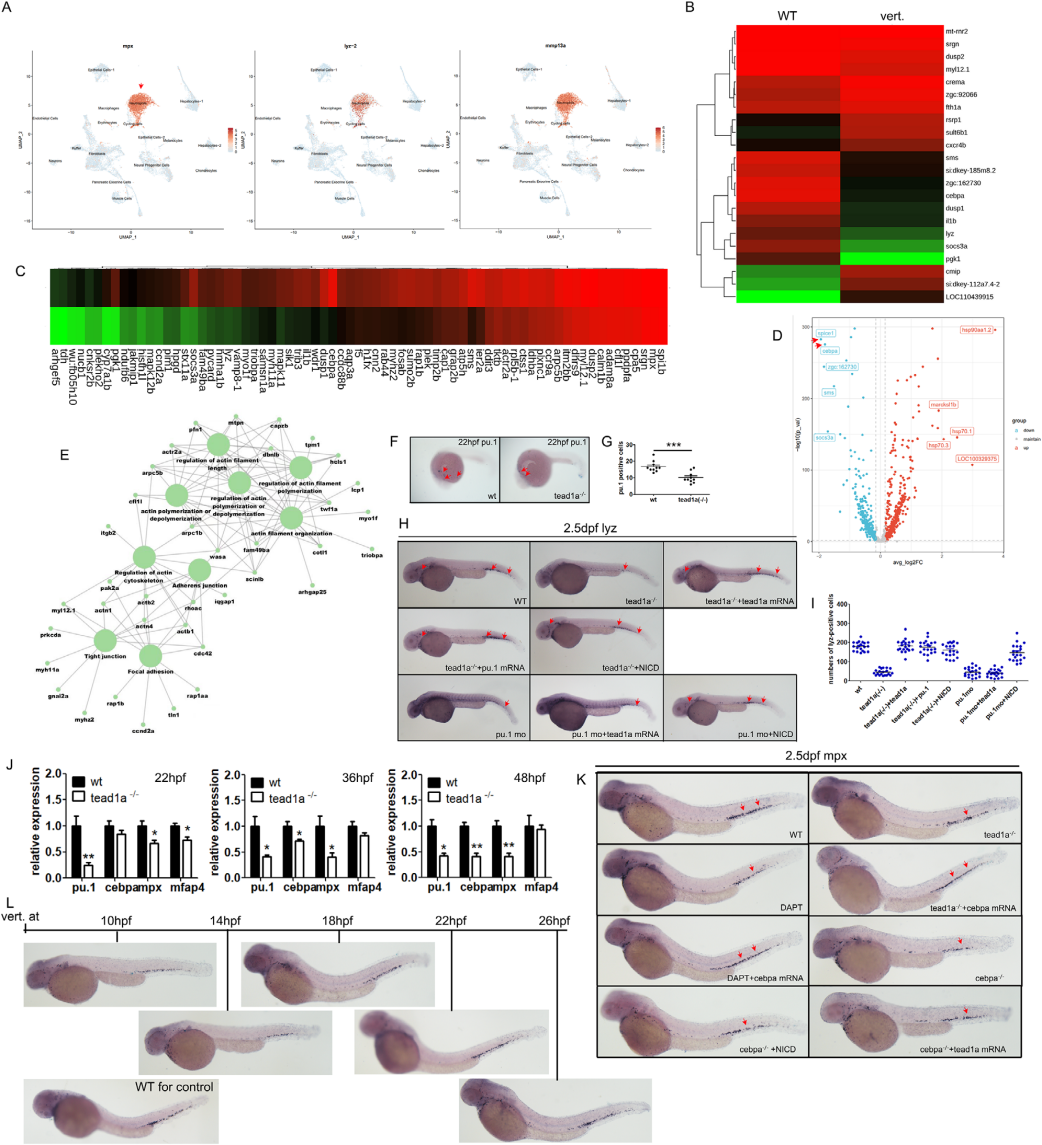
Tead1a improves neutrophil cell differentiation through TEAD/YAP‐notch‐spi1/cebpα axis and time window. A–E) scRNA‐seq data and analysis results of whole embryo upon verteporfin treatment. (A) scRNA‐seq data showing the sorting of eGFP positive cells are neutrocytes, here are the expression plots of selected markers for neutrophils: mpx, liz, and mmp13a. (B) Heatmap of the top 60 markers in neutrophils (complete list available in Data S3, Supporting Information, log fold‐change threshold = 0.25 and p‐value <0.05, out of the 1463 genes, only 461 genes with positive fold change were selected). (C) Heatmap of the 72 high‐ranking downregulated reported genes in neutrophils (complete list available in Data S4, Supporting Information, log fold‐change threshold = 0.4 and p‐value <0.001). (D) Volcano plot of significantly differently expressed genes in neutrophils (complete list available in Data S4, Supporting Information, log fold‐change threshold = 0.4 and p‐value <0.001). (E) The network analysis of the pathways associated with downregulated genes in the neutrophil subcluster. F,G) WISH analysis of *pu.1* expression at 22 hpf. H,I) The rescue effect of *pu.1* and NICD on the neutropenia caused by *tead1a* mutation. J) Whole‐embryo qPCR analysis of zebrafish at 22 hpf, 36 hpf and 48 hpf. K) The rescue effect of *cebpα* on the neutropenia caused by DAPT or *tead1a* mutation. L) Verteporfin caused the phenotype of neutropenia only before the time window of 10–18 hpf. Ver. For verteporfin. Error bars represent the mean ± standard deviation (SD) of at least 15–40 embryos or larvae from three independent experiments. Statistical significance was calculated using the Student's *t*‐test. *, *p* <0.05; **, *p* <0.01; ***, *p* < 0.001; NS, not significant.

Our results thus far have shown that blocking the TEAD/YAP pathway with verteporfin inhibited granulopoiesis in zebrafish, which is consistent with the granulocyte deficiency phenotype caused by mutations in *tead1a*. After that, we applied single‐cell sequencing to examine zebrafish larvae treated with verteporfin at 72 hpf to discuss the ultimate site of action of this intervention on neutrophils. We performed cell clustering using single‐cell sequencing, and we used the transgenic fish line Tg(*mpx*:eGFP) instead of WT to enhance the proportion of neutrophil selection. We named each cluster based on cell‐type origins and based on known lineage marker genes (Figure S3A, Supporting Information). The whole sequencing and analysis results were in Data S3, Supporting Information “single‐cell sequencing of all clusters upon vert training”. Due to the low proportion of neutrophils in the whole embryo, which can affect the sequencing analysis of the neutrophil subcluster, we first sorted cells with *mpx*‐eGFP to ensure that the number of neutrophils reached 1000–2000. Subsequently, the sorted neutrophils were mixed with other randomly obtained whole‐embryo cells for sequencing. We initially identified a subpopulation of neutrophils based on the high expression of *mpx*, *lyz*, and *matrix metalloproteinase 13a (mmp13a)* (**Figure** **7**A).

Next, we analyzed genes that were differentially expressed in neutrophils after verteporfin training. We found 131 genes were significantly upregulated, and 116 were downregulated upon verteporfin training (Figure 7B–D; Data S4, Supporting Information “cluster1 neutrophils upon vert training”). Our transcriptomic analysis revealed that markers highly expressed in neutrophils, such as *lyz*, *cebpα*, and *spi1*, were downregulated. The results of the volcano plot for differential gene expression indicate that *spice1* and *cebpα* may be the key factors in the TEAD/YAP‐notch1 axis (Figure 7D). We next performed a network analysis of the pathways associated with downregulated genes in the neutrophil subcluster, the result certified the dysplasia and dysfunction of neutrophils instead of merely decreased quantity upon verteporfin training (Figure 7E). In the network analysis, the destruction of TEAD or TEAD/YAP binding led to main dysfunctions as follows: regulation of actin cytoskeleton, actin filament organization, regulation of actin filament polymerization, actin polymerization or depolymerization, regulation of actin filament length, tight junction, focal adhesion, and adherens junction (Figure 7E).

In our study, *pu.1* was downregulated in *tead1a* ^‐/‐^ as early as 22 hpf (Figure 7F). We constructed the *pu.1* and *cebpα* mRNA‐PCS2 plasmid, prepared the *pu.1* MO and *cebpα* mutation zebrafish line, and then performed embryonic microinjections in the zebrafish model to further WISH analysis. The results revealed that suppressed granulopoiesis in zebrafish could be rescued by *tead1a* mRNA, *pu.1* mRNA, or NICD. In contrast, the neutropenia caused by *pu.1* MO sequence could not be rescued by *tead1a* mRNA and could be partially rescued by NICD fragments. These results indicate that the promotive effect of *tead1a* on zebrafish embryonic granulopoiesis depends on *pu.1* and *notch1* being realized. In contrast, the promotive effect of *pu.1* on granulopoiesis does not depend on *tead1a* and is partially dependent on NICD or the high expression of the notch1 signaling pathway it represents. The promotive effect of *notch1* on granulopoiesis does not depend on *tead1a* and is not entirely dependent on *pu.1* (Figure 7H,I). We inserted the sequence of fragments nearest to the transcription start site (−1.7 kb) of the zebrafish *pu.1* promoter into a luciferase reporter vector.^[^ ^48^ ^]^ None of the luciferase expressions changed significantly when co‐transfected with either tead1a or NICD sequence in HEK293T cells (Figure S3B, Supporting Information).

In whole‐embryo qPCR analysis, we detected from 22 hpf to 48 hpf, the expression difference of *pu.1* was reducing between WT and *tead1a* ^‐/‐^, while the expression differences of *cebpα* and *mpx* were somewhat enlarging (Figure 7J). Thus, we continued to explore the role of *cebpα* using WISH analysis. As a result, *cebpα* can significantly rescue the neutropenia caused by *tead1a* mutation or DAPT, while the neutropenia caused by *cebpα* mutation could be rescued by neither *tead1a* mRNA nor NICD, implying a terminal and essential effect of *cebpα* (Figure 7K).

To our surprise, both the inhibitory effect of verteporfin on neutrophils and the rescuing effect of Notch signaling pathway activation on the neutrophil defects in the Tead1a‐mutated fish line were only effective when applied before 10–14 hpf, with significantly reduced effects at 18–22 hpf, and no effect at all after 26 h (Figure 7L). This result led us to believe that the regulation of neutrophils by the TEAD/YAP‐Notch1 signaling pathway does not act on mature neutrophil subpopulations and may even precede the emergence of hemogenic endothelium (HE). However, confirming this hypothesis requires further specific retrospective studies.

In summary, our study innovatively discovered that the transcription factor TEAD1a, originally thought to function only during the embryonic development gastrulation stage, significantly promotes neutrophil development and maturation through the TEAD/YAP‐notch1‐pu.1/cebpα signaling axis. Moreover, to our delight, we found that this early embryonic regulatory effect is exerted only during the early stages of embryonic development, thereby locking the directed development of certain neural crest‐derived neutrophil‐related progenitors in the early stages of embryogenesis. Our research deepens our understanding of how transcription factors regulate signal pathways to modulate neutrophil development.

## 3 Discussion

Transcriptional priming is an epigenetic or transcriptional pre‐patterning process that determines subsequent differentiation trajectories, which has been established during or prior to the formation of HSCs or HSPCs.^[^ ^14^ ^]^ For example, short‐term exposure to nicotinamide riboside (NR) alters the transcriptional priming of the HSCs toward lymphoid lineages in mice model.^[^ ^49^ ^]^ Mutations in DNA methylation‐associated genes such as ten‐eleven translocation 2 (TET2) and DNA methyltransferase 3A (DNMT3A)disrupt the differentiation of murine HSPCs. Specifically, Tet2 deletion results in increased frequency of myelomonocytic progenitors and reduced erythroid progenitor output, whereas Dnmt3a loss induces opposite differentiation skews, and these lineage biases are traceable to transcriptional priming biases in uncommitted HSCs.^[^ ^50^ ^]^

In the present study, we determined that *tead1a* first stimulates myeloid progenitor cells through the TEAD/YAP‐notch1 signaling pathway at exceedingly early and key developmental stages in zebrafish by the initiation of transcriptional priming. Then, spi1 was stimulated and continued to promote neutrophil cell fate. Zebrafish *tead1a* acts as an original and novel player in neutrophil cell proliferation and fate decisions, with the critical window occurring at the exceedingly early stage of hematopoiesis.

Neutrophil deficiency‐related diseases pose considerable clinical risks, and the only conservative treatment currently available is G‐CSF therapy, which has poor clinical outcomes and substantial side effects, potentially leading to hematopoietic system malignancies.^[^ ^3^, ^51^, ^52^, ^53^ ^]^ Neutrophils promoted by G‐CSF are defective; even if their numbers are normal, they can be developmentally abnormal and functionally blunted.^[^ ^4^ ^]^ However, no specific activator has promoted neutrophil development earlier than G‐CSF, thereby fundamentally improving neutrophil developmental defects and promoting effective neutrophil proliferation as a treatment method. In our research, *tead1a* exhibited a promoting effect on neutrophils at an earlier stage of granulocytic development, and its mechanism of action was independent of G‐CSF (csf3r in zebrafish).^[^ ^53^, ^54^ ^]^ Moreover, there have been no reports of myeloid malignancies with high expression of the *tead1* gene, which theoretically holds major research value and clinical implications.

Nowadays, TEAD proteins have been reported to exhibit heightened expression in the early stages of zebrafish embryogenesis during gastrulation, facilitating the differentiation and development of HSCs.^[^ ^18^ ^]^ During several developmental stages of murine embryonic stem cells cultured in vitro, TEAD was encoded at the stage of epithelial hematopoietic differentiation. The functional block of TEAD/YAP by verteporfin in HPs makes little difference in their number or survival. In contrast, when it happens in embryoid bodies or AGM‐derived Flk1^+^endothelium, verteporfin inhibits the production of HPs. These results indicate a requirement for YAP/TEAD in the endothelium before EHT.^[^ ^18^ ^]^ TEAD/YAP may be essential in the HE stage and activated by embryonic blood flow. In studying human CD34^+^ endothelial cells and zebrafish embryos, the cyclic stretching of the endothelium from embryonic blood flow activates YAP. This leads to the expression of Runx1 and the production of HSPCs.^[^ ^55^ ^]^ In our research, although the time point of action for TEAD/YAP must also precede the formation of vascular endothelium, its primary role is in neutrophils, suggesting that its stimulatory effect on neutrophils occurs even earlier than that of G‐CSF.

In our research, after blocking TEAD/YAP in myeloid precursor cells, we identified *notch1* as a key effector molecule among numerous downstream signaling molecules. Using NICD to mimic Notch signaling activation can rescue the neutrophil deficiency phenotype induced by TEAD/YAP blockade, thereby indirectly confirming the importance of Notch signaling. Notch is a transmembrane receptor, and its signaling is established through cell‐cell contact. Notch receptors in a signal‐receiving cell bind to JAGGED and DELTA ligands in a signal‐emitting cell and undergo two cleavage events before finally releasing NICD that translocate to the nucleus to modulate the transcription of Notch target genes.^[^ ^56^ ^]^ There is no direct evidence to prove that Notch is a downstream signaling molecule of TEAD/YAP. However, it has been reported that YAP can upregulate the Notch ligand JAGGED.^[^ ^57^ ^]^ Thus, it is reasonable to suspect that TEAD1 may affect NICD and its downstream target genes through this effector or similar other effects, and its action may persist in myeloid precursor cells. Further research is needed on whether they act on the same cell subpopulation and then become dormant as the cells develop, or act on different developmental stage cell subpopulations.

Notch is also essential for the early stages of hematopoiesis. Activation of Notch is required for hemogenic endothelium formation in the DA.^[^ ^58^ ^]^ In zebrafish, Notch1 receptors are expressed in the DA during the window of HSPC emergence and support the emergence of nearly the earliest markers of HSPCs, runx1.^[^ ^6^, ^59^ ^]^ Although the absence of Notch‐runx1 signaling despairs the form of HSPCs, this does not harm erythro‐myeloid progenitors (EMPs), which do not express the Notch receptor at all.^[^ ^60^, ^61^ ^]^ Furthermore, Notch signaling may contribute to the incomplete elimination of leukemic cells, helping their proliferation while inhibiting their differentiation.^[^ ^62^ ^]^ Notch1 activation enhances proliferation and delays the granulocytic differentiation of 32D cl3cells.^[^ ^63^ ^]^ Recombinant Jagged1 (JAG1) protein, theoretically as a downstream molecule of TEAD/YAP, can merely promote myeloid notch signaling under special conditions.^[^ ^64^ ^]^

Our study supports these studies that YAP/TEAD‐notch1 is essential in the endothelium before EHT‐ and HE‐induced HSPCs, even though their final target is neutrophils. The reason is that the time window in either *tead1* (10‐18 hpf) is considerably early.

We were intrigued to find that the most considerable impact was on neutrophils, despite our initial signaling pathways affecting the entire HE and HSPCs. Overexpression of TEAD had little effect on erythroid progenitor cells due to the early activation of HSCs and the subsequent suppression of *gata1*. Consistent with this, TEAD deficiency resulted in no significant change in red blood cell numbers but a reduction in gata1 signaling and a subsequent decrease in a series of erythroid genes. Thus, the further the differentiation into erythroid cells, the more evident the inhibitory effect of TEAD1 on a series of key genes in red blood cells becomes. Macrophages were almost unaffected due to the replenishment of macrophages by EMPs. *Pu.1* is crucial for macrophage development, and our single‐cell sequencing confirmed that the expression of *pu.1* in the macrophage population dropped to about one‐tenth of its original level. In contrast, genes such as *mfap4* and *csf1r* within macrophages remain unchanged. This raises the question of whether the formation and number of macrophages are independent of *pu.1*, which requires further research.

It is worth saying, that due to the drastic reduction of neutrophils in zebrafish embryos after verteporfin treatment, and the fact that a low proportion of neutrophils in the whole embryo can affect the sequencing analysis of neutrophil subpopulations, we first sorted cells with GFP fluorescence to ensure that the number of neutrophils reached 1000–2000. Subsequently, the sorted neutrophils were mixed with other randomly obtained whole‐embryo cells for sequencing. Therefore, the proportions of these cell subpopulations do not represent their actual proportions in the whole embryo. However, the proportions of cell subpopulations other than neutrophil subpopulations can roughly reflect their actual proportions.

The current work has multiple limitations, based on which we proposed future plans. First, we need to deepen our research for molecular mechanisms, for example, the epigenetic regulation via TEAD binding motifs: We need scATAC‐seq to interrogate occupancy of the TEAD consensus motif (GGAATG) within regulatory elements of *notch1* promoters, *spi1* promoters, and Cebpα enhancers. Another example, functional validation of protein‐DNA interactions: We plan to express of HA‐tagged TEAD1a and HA‐tagged NICD in murine myeloid progenitor 32D cl3 cells, and propose chromatin immunoprecipitation sequencing (ChIP‐seq) using anti‐HA antibodies to genome‐widely map TEAD1a or NICD binding loci. Second, we observed that TEAD1a did not manifest overt erythroid suppressive effects at 22 hpf, which was potentially attributable to its role in promoting the proliferation of most HSPCs in the primitive hematopoietic stage, which in turn generates an increased number of erythroid precursor cells, thereby obscuring its impact on subsequent erythroid developmental processes and we need to make this clear. Not but not the least, although extensive research focus on the Hippo pathway has led to the development of numerous small‐molecule inhibitors targeting the TEAD/YAP interaction and downstream signaling,^[^ ^65^, ^66^, ^67^ ^]^ no clinically approved therapeutics exist that directly activate TEAD/YAP signaling to treat human diseases. Substantial challenges persist in translating TEAD/YAP pathway activation into clinical practice, particularly for promoting granulocytic hematopoiesis. Future success hinges on elucidating the physiological roles of TEAD/YAP in homeostasis and developing targeted agonist modalities, which are critical for advancing clinical applications in this field.

Our research only explores one signaling pathway that affects hematopoietic development. The most exciting finding in our study is that even factors with major roles at the HSPC stage also exhibit a clear bias toward later cell subpopulations. This suggests that hematopoietic development may begin to form directed hematopoietic cell subpopulations exceedingly early (at the time of HSPC formation), and the differentiation programs for erythroid, neutrophil, and macrophage subpopulations may be partially established.

In summary, our study initially discovered the essential and special role of *tead1a* in stimulating neutrophil development, which begins at an extremely early stage and relies on TEAD/YAP‐Notch‐Spi1/Cebpα axis and an early developmental time window. This study can not only improve the understanding of the intricately orchestrated nature of embryonic development and related stage‐specific regulation, but also facilitate promising therapeutic development for clinical benefit.

## 4 Experimental Section

### Maintenance and Generation of Zebrafish

The zebrafish strain individuals were raised, bred, and maintained under standard conditions at 28.5 ± 0.5 °C on a 12 h light:12 h dark cycle. The embryos were grown in egg water containing 60 µg mL^−1^ sea salt and 0.2% methylene blue, N‐Phenylthiourea (PTU; Sigma–Aldrich; 0.045%) was used within 12 hpf.

For the generation of CRISPR/Cas9‐mediated *tead1a* mutant zebrafish, guide RNA targeting exon 1 of *tead1a* was designed using an online tool, ZiFiT Targeter software(http://zifit.partners.org/ZiFiT), and then injected into one‐cell stage zebrafish embryos together with Cas9 mRNA synthesized using mMESSAGE mMACHINE ^TM^ T7 Transcription synthesis Kit (Ambion). The injected F0 founder embryos were raised, and after genotyping, they were outcrossed with wild‐type zebrafish, and published transgenic lines included Tg(*gata1*: DsRed), Tg(*mpx*:eEGFP), and Tg(*mpeg1*:eEGFP), respectively. F1 embryos were raised, in‐crossed after genotyping. The lines of *tead1a^‐/‐^*, *tead1a ^‐/‐^* Tg(*gata1*: DsRed), *tead1a ^‐/‐^* Tg(*mpx*:eEGFP), *tead1a ^‐/‐^* Tg(*mpeg1*:eEGFP), were then established and used. The genotyping PCR primers were as follows: forward 5′‐ GCTGCTTCCCAGGTCAGT‐3′; reverse 5′‐ TGGGATAAATTTGTCGATTCCAA‐3′.

### Plasmid Construction

Zebrafish genes and mutants of 0.7–1.2 kb sequences were amplified from the reverse transcription products of 0–7dpf zebrafish embryo mix and inserted into the FLAG‐pre‐tagged PCS2^+^ vector (using the restriction enzymes of EcoR 1, Xho 1 or Not 1 downstream of the SP6 promoter) with their respective primers. The Flag‐pre‐tagged PCS2^+^ vector was modified by our laboratory in advance by inserting the FLAG sequence (without the stop codon) into the upstream of the EcoR1 restriction enzyme site. The ≈2 kb upstream sequence of zebrafish *gata1* open reading frame was amplified with PCR using the WT genes as templates and ligated into the Kpn 1 and Sac 1 sites of the PGL3^+^ basic vector (Promega) to generate the luciferase reporter (Table S1, Supporting Information). We conducted PCR using plasmids containing the coding sequence of WT gene (*tead1a*) as templates and inserted the amplified mutant sequences into the FLAG‐pre‐tagged PCS2^+^ vector (using the restriction enzymes of EcoR 1 and Xho 1 downstream the SP6 promoter) (Table S2, Supporting Information).

### Morpholinos and mRNA Microinjection

Morpholinos (MOs) and mRNA microinjection are extremely valuable research tools used to study embryo development in vivo. While MOs mimic gene knock‐down, mRNA microinjection simulates gene expression. The MOs were designed using Genetools (www.genetools.com)^[^ ^30^ ^]^ and the sequences are as follows: *tead1a* MO: 5′‐CATGGCAATGGATGTGATCTCAGA‐3′; *pu.1*/*spi1* MO: 5′‐GATATACTGATACTCCATTGGTGGT‐3′. Full‐length mRNA samples were all synthesized using an SP6 kit (Life Technologies) from plasmid products after endonuclease digestion. After preparation, microinjection of mRNA was conducted into the yolk sac site close to the single cell of embryos at the 1‐cell stage (before 0.5 hpf). The concentrations of mRNA samples used for microinjection were ≈40–120 ng µL^−1^, depending on experimental conditions.

### Drug Treatment

Zebrafish embryos were incubated in 3–5 µm verteporfin at the respective embryonic stage using the Dimethyl Sulfoxide (DMSO) 3–5 µL mL^−1^ egg water solution for embryo treatment. Zebrafish embryos were exposed to DAPT with a microinjection concentration of 300 µm at the 1‐cell stage.

### Flow Cytometry and Cell Sorting

Whole embryos were collected from two zebrafish lines, normal Tg(mpx: eGFP) and *tead1a* ^‐/‐^//Tg(mpx: eGFP), and then carefully dissected and maintained in 0.9 × phosphate‐buffered saline (PBS) with 2% fetal bovine serum on ice before being placed on a Falcon nylon cell strainer (Beckton Dickinson); gravity was then used to obtain a single‐cell suspension. For eGFP^+^ myeloid cells or neutrophils sorting, at least 400 embryos per group, one time and up to four times, were needed to separate the needed cells for further analysis, including quantitative PCR, RNA sequencing, and single‐cell RNA sequencing.

### Staining of Embryos and/or Larvae

Embryos and/or larvae were fixed overnight and stained by Sudan Black as before.^[^ ^29^ ^]^ Embryos and/or larvae were stained with O‐dianisidine according to a previous study.^[^ ^68^ ^]^ In brief, the working concentration of the O‐dianisidine solution was 0.6 mg mL^−1^ and contained 0.01 m sodium acetate (pH 5), 40% ethanol, and 0.8% hydrogen peroxide. Finally, fresh embryos were stained and gently shaken in the dark for 30 min at room temperature. Embryos and/or larvae were stained with Neutral Red following the routine procedures.^[^ ^69^ ^]^

### Extraction of DNA and RNA and Quantitative PCR

For cell‐sorting qPCR, eGFP^+^ cells were sorted from normal Tg(*mpx*: eGFP) and *tead1a* ^‐/‐^ Tg(mpx: eGFP) at 72 hpf or another phase. The embryos were selected, cleaned, and cut into pieces for whole‐embryo qPCR. The total RNA was extracted using TRIzol reagent (Life Technologies) and reverse transcribed into cDNA using a Revert Aid First Strand cDNA Synthesis Kit (Thermo). Amplification products were synthesized using SYBR Green Real‐time PCR Master Mix (TOYOBO). All primers for quantitative PCR were collected in Table S3 (Supporting Information).

### Whole Mount In Situ Hybridization (WISH)

Whole‐mount mRNA in situ hybridization was conducted as described previously.^[^ ^70^ ^]^ Digoxigenin (DIG)‐labeled RNA probes were transcribed with T7, T3 or SP6 polymerase (Ambion). Probes labeled with DIG (Roche) were detected using anti‐digoxigenin Fab fragment antibody (Roche) with 5‐bromo‐4‐chloro‐3‐indolyl phosphate/nitroblue tetrazolium (BCIP/NBT) staining (Vector Laboratories). The indicated primers for WISH probes were shown in Table S4 (Supporting Information).

### RNA‐Seq of Zebrafish Kidney Marrow and 32d cl3 Cell Line

Zebrafish kidney marrow was dissected from 6‐month‐old fish after being anesthetized in ice, with at least 15 fish per group. Then, the kidney marrow was made into single‐cell suspensions in 20 mL of 4 °C PBS and then resolved in TRIzol reagent (Life Technologies). The 32D cl3 cell line was cleaned and collected in a TRIzol reagent. Total RNA was extracted for RNA sequencing. RNA libraries were constructed using the MGI Easy RNA Library Prep Set according to the manufacturer's protocol. Libraries were sequenced using the BGISEQ500 platforms.

### Cell Transfection and Luciferase Reporter Assay

HEK293T cells were cultured in Dulbecco's modified eagle medium (DMEM) (Gibco) with 10% fetal bovine serum (FBS) (Gibco) at 37 °C under 5% CO_2_. Plasmid samples were transfected into cells using Effectene Transfection Reagent (QIAGEN). HEK293T cells were seeded at a density of ≈4 × 10⁴ cells/well in 24‐well plates using 500 µL of DMEM. Then we mixed 200 ng plasmid DNA (dissolved in TE buffer) with Buffer EC to a final volume of 60 µL. Then 1.6 µL Enhancer was added, followed by vortexing for 1 s and brief centrifugation. The mixture was incubated for 2–5 min to facilitate DNA condensation. (DNA: Enhancer mass ratio 1:8). Then, 5 µL Effectene Transfection Reagent was added and mixed and incubated for 5 min. Then cells were washed once with PBS, and 350 µL fresh complete medium was added. The transfection complexes were diluted in 350 µL fresh medium and added. The cells were then harvested 40–48 h before protein extraction, or luciferase reporter assays were conducted with the Dual Luciferase Reporter Assay Kit (Promega), as previously described.^[^ ^70^ ^]^

### Single‐Cell RNA‐Seq Analysis

The scRNAseq datasets were preprocessed using Seurat in R (https://satijalab.org/seurat/).^[^ ^71^ ^]^ Cells with more than 10% of mitochondrial gene fraction, less than 300 detected genes, more than 7500 genes or less than 500 UMIs were discarded. Data analysis results were provided in the Supporting Information.^[^ ^72^ ^]^ A total of 18367 cells passed the quality control, and 47.395 genes were analyzed. Marker genes for each cluster were inspected, and cluster identities were determined based on previously reported lineage‐specific marker genes in zebrafish.^[^ ^14^, ^73^, ^74^, ^75^, ^76^, ^77^, ^78^, ^79^, ^80^, ^81^ ^]^ Differentially expressed genes were analyzed based on their known functions, and heatmaps were constructed using the OmicShare tools platform (https://www.omicshare.com/tools/).^[^ ^82^ ^]^

### Statistical Analysis

All experiments were performed with at least three independent biological replicates using embryos from different mating pairs. Data are presented as mean ± standard deviation (SD) of biological replicates, and statistical analyses were performed accordingly. Statistical significance was determined by a two‐tailed unpaired *t*‐test. For survival analysis, Kaplan‐Meier survival curves were analyzed, and statistical significances were calculated using GraphPad Prism 6.0.

Analysis of WISH and other embryo staining images involved at least 15–40 embryos from three independent experiments. Positive areas of gene expression in every embryo involved in WISH analysis were selected and calculated as a proportion of the total captured embryo area using Photoshop_CS5, and the results were calculated using GraphPad Prism 6.0. Outliers were removed using the ROUT method, with Q = 1%. Statistical significance was determined with a *p*‐value of 0.05.

### Ethics Approval and Consent to Participate

All animal experiments were approved by the Institutional Animal Care and Use Committee (IACUC) of Shanghai Jiao Tong University and South China University of Technology.

## Conflict of Interest

The authors declare no conflict of interest.

## Author Contributions

W.Y., Y.R., and W.P. contributed equally to this work. W.Y., X.B., and Y.R. designed research; W. Y., X. B., L. X., and Y. H. performed research; Y.R., W.P., and H.H. analyzed data; and W.Y., X.B., H.H., and Z.Y. conceived the project and wrote the paper.

## Supporting information



Supporting Information

Supplemental Table 1

Supporting csv

Supplementary Dataset

## Data Availability

The data that support the findings of this study are available from the corresponding author upon reasonable request.
