# Supplementary material for: Tead1a Initiates Transcriptional Priming Through the TEAD1a/YAP‐Notch1‐Spi1/Cebpα Axis to Promote Neutrophil Fate
Source: Adv Sci (Weinh). 2025 Aug 28;12(41):e05441. doi: 10.1002/advs.202505441 (PMC12591127; doi:10.1002/advs.202505441)
Supplement: Supplementary file 1 — Supporting Information [file ADVS-12-e05441-s003.docx]

**Tead1a initiates transcriptional priming through the TEAD1a/YAP-Notch1-Spi1/Cebpα axis to promote neutrophil fate**

**Supplemental Tables**

**Supplemental Table 1. Primers used for PCS2^+^ plasmid construction of segments of wild-type gene CDS sequences and promoter-PGL3 plasmid construction. The plasmid of NICD- PCS2^+^ is from the lab. Pu.1 promoter was from the lab which we reported before (**[**1**](#_ENREF_1)**).**

| **gene** | **Primer sequence (5’-3’)** |
| --- | --- |
| **Tead1a** | **SENSE: 5’GGAATTC****GATCCCAGCAGCTGGAGCCCTGAC 3’** |
|  | **ANTISENSE: 5’** **CCTCGAGTCAATCTTTGACCAGTCTGTAG 3’** |
| **Yap1** | **SENSE: 5’** **GGAATTCGATCCGAACCAGCACAACCCTCCAG 3’** |
|  | **ANTISENSE: 5’** **CCTCGAGCTATAGCCAGGTTAGAAAGTTCTCCTTGTCG 3’** |
| **Gata1-promoter** | **SENSE: 5’ GGGGTACCCTTTTGCATTTTCATTCGCTT 3’** |
|  | **ANTISENSE: 5’ CGAGCTCCAGGTGCAATGGTATAACACA 3’** |
| **Ctgfa** | **SENSE: 5’** **GGAATTCTTTTCTGGAATGACTCAAAGT 3’** |
|  | **ANTISENSE: 5’** **CCTCGAGTCACGCCATGTCGCCAACCATCTTCTTG 3’** |
| **Cyr61** | **SENSE: 5’** **GGAATTCTTTGCTTGGGCTGTTATCGTCATC 3’** |
|  | **ANTISENSE: 5’** **CCTCGAGTCAGTCTGTGAACTTGGGATGTCGTTG 3’** |
| **Cdk6** | **SENSE: 5’** **GGAATTCGACAAAGAGAGCTCGACCAGCAATATGAGC 3’** |
|  | **ANTISENSE: 5’** **GCGGCCGCTTAGGCGGCTCTCTCCTCCAGGGAGGGC 3’** |
| **Pu.1** | **SENSE: 5’** **GGAATTCCTGCATCCGTACAGAATGGAG 3’** |
|  | **ANTISENSE: 5’** **CCTCGAGTTACATGTAATGCTTTCTGTCTGTG 3’** |
| **cebpα** | **SENSE: 5’** **GGAATTCGAGCAAGCAAACCTCTACGAG 3’** |
|  | **ANTISENSE: 5’** **CCTCGAGTTAAGCGCAGTTGCCCATGGC 3’** |

**Supplemental Table 2. Primers used for pcs2+ mutant gene plasmid construction.**

| **gene** | **Primer sequence (5’-3’)** |
| --- | --- |
| **Tead1a dYBD** | **SENSE: 5’ GGAATTCGATCCCAGCAGCTGGAGCCCTGAC 3’** |
|  | **ANTISENSE: 5’** **CCTCGAGTCAGGGCGATCGGCTGATCTTGTA 3’** |
| **Tead1a dDBD** | **SENSE: 5’** **GGAATTCTACATCAAACTGCGAACGGGCAAG 3’** |
|  | **ANTISENSE: 5’** **CCTCGAGTCAATCTTTGACCAGTCTGTAG 3’** |

**Supplemental Table 3. Primers used for q-PCR analysis in zebrafish.**

| **gene** | **Primer sequence (5’-3’)** |
| --- | --- |
| **Fli1** | **SENSE: 5’ GGCTCTCCAACAGTGGTCTC 3’** |
|  | **ANTISENSE: 5’** **GAACTGCCACAGCTGGATCT 3’** |
| **Gata2** | **SENSE: 5’** **CGCTTCCAGCTTCACACCTA 3’** |
|  | **ANTISENSE: 5’** **GCGCTTTGGCTTGATAAGGG 3’** |
| **Scl** | **SENSE: 5’** **TAGCAATCGAGTCAAGCGCA 3’** |
|  | **ANTISENSE: 5’** **CAATTTTGGGCTGCGAACCA 3’** |
| **Lmo2** | **SENSE: 5’** **GGGACGCAGGCTTTACTACA 3’** |
|  | **ANTISENSE: 5’** **GCACACGCATGGTCATTTCA 3’** |
| **Gata1** | **SENSE: 5’AGCGCTCTATTCAACTGGGG 3’** |
|  | **ANTISENSE: 5’CCAGCACGTTTGCTGACAAT 3’** |
| **Hbαe1.1** | **SENSE: 5’CCAGGATGTTGATTGTCTAC 3’** |
|  | **ANTISENSE: 5’CAGTCTTGCCGTGTTTC 3’** |
| **Mpx** | **SENSE: 5’** **GCTATACCAGGTTATAATGCATG 3’** |
|  | **ANTISENSE: 5’** **CCACAACCTATCGCCATCTCG 3’** |
| **Mfap4** | **SENSE: 5’** **GGATGGACGGTGATTCAGAG 3’** |
|  | **ANTISENSE: 5’** **CAGATAAAGAGTCGCCTGCT 3’** |
| **L-plastin** | **SENSE: 5’** **AGAAGAAGCTGACGCCCTTC 3’** |
|  | **ANTISENSE: 5’** **GCTGATCTCGATGTCTGCGA 3’** |
| **Flk1** | **SENSE: 5’** **TAGTGTGTGAAGTGTCCGGC 3’** |
|  | **ANTISENSE: 5’** **AGCCTCACTGGATTCACAGC 3’** |
| **Runx1** | **SENSE: 5’** **GAACTTCCTCTGCTCCGTCC 3’** |
|  | **ANTISENSE: 5’** **TATCACCAAGGGCAACCACC 3’** |
| **Lyz** | **SENSE: 5’** **GCCTGTTCAGACTTGCTTAACG 3’** |
|  | **ANTISENSE: 5’ CAGGCTCGGAGGCTTTGTTTG 3’** |
| **Csf1r** | **SENSE: 5’** **CAGACGCAGACAGTGGAGTT 3’** |
|  | **ANTISENSE: 5’** **TGACCGACCAGGTTAAAGGC 3’** |
| **Spi1** | **SENSE: 5’** **GACAGTCAGAACGATCACTCTT 3’** |
|  | **ANTISENSE: 5’** **GGAGAGGAGATGGCTGGACG 3’** |
| **Mpeg1** | **SENSE: 5’** **CTCCACAGAAAACCAGCGCA 3’** |
|  | **ANTISENSE: 5’** **CGTCAGCGATTTCTTCTGCC 3’** |
| **C-myb** | **SENSE: 5’** **TTCATCCGTCAGACACCTGC 3’** |
|  | **ANTISENSE: 5’** **AGTGCTTTCTGGGAGCAGAC 3’** |
| **Hbαe3** | **SENSE: 5’CCTAAGCCCCAACTCTC 3’** |
|  | **ANTISENSE: 5’CTCCCTTCAGGTCATCC 3’** |
| **Cebpα** | **SENSE: 5’** **TGAAGATTGGCGATCGAGGG 3’** |
|  | **ANTISENSE: 5’** **ATTTTCGCCTTGTCCCGACT 3’** |
| **Il-1β** | **SENSE: 5’** **CTGAAATGATGGCATGCGGG 3’** |
|  | **ANTISENSE: 5’** **TGCAAGCGGATCTGAACAGT 3’** |
| **Il-6** | **SENSE: 5’** **GGCATTTGAAGGGGTCAGGA 3’** |
|  | **ANTISENSE: 5’** **CGTTCACCAGGACTGAGGTC 3’** |

**Supplemental Table 4. Primers used for WISH molecular probe construction.**

**Probes of gata2, fli1, scl, lmo2, gata1, hbae1.1, mpx, mfap4, L-plastin, c-myb, apoe, pu.1 and rag1 were offered in our lab (**[**1-5**](#_ENREF_1)**). Probe of hbae3 was constructed as follow:**

| **gene** | **Primer sequence (5’-3’)** |
| --- | --- |
| **Hbae3** | **SENSE: 5’CTCTTCACAGCTTT 3’** |
|  | **ANTISENSE: 5’TTAGCGGTACTTCTCGGA 3’** |

1. Wang, L., Gao, S., Wang, H., Xue, C., Liu, X., Yuan, H., Wang, Z., Chen, S., Chen, Z., de Thé, H. *et al.* (2020) Interferon regulatory factor 2 binding protein 2b regulates neutrophil versus macrophage fate during zebrafish definitive myelopoiesis. *Haematologica*, **105**, 325-337.

2. Chen, H., Wang, Z., Yu, S., Han, X., Deng, Y., Wang, F., Chen, Y., Liu, X., Zhou, J., Zhu, J. *et al.* (2021) 3,3',5-Triiodothyroacetic acid (TRIAC) induces embryonic ζ-globin expression via thyroid hormone receptor α. *Journal of hematology & oncology*, **14**, 99.

3. Han, X., He, W., Liang, D., Liu, X., Zhou, J., de Thé, H., Zhu, J. and Yuan, H. (2024) Creg1 Regulates Erythroid Development via TGF-β/Smad2-Klf1 Axis in Zebrafish. *Advanced science (Weinheim, Baden-Wurttemberg, Germany)*, **11**, e2402804.

4. Yang, R.M., Song, S.Y., Wu, F.Y., Yang, R.F., Shen, Y.T., Tu, P.H., Wang, Z., Zhang, J.X., Cheng, F., Gao, G.Q. *et al.* (2023) Myeloid cells interact with a subset of thyrocytes to promote their migration and follicle formation through NF-κB. *Nature communications*, **14**, 8082.

5. Deng, Y., Wang, H., Liu, X., Yuan, H., Xu, J., de Thé, H., Zhou, J. and Zhu, J. (2022) Zbtb14 regulates monocyte and macrophage development through inhibiting pu.1 expression in zebrafish. *eLife*, **11**.

Supplemental figure 1. Hematopoietic gene expression changes induced by *tead1a* MO. (A, C) WISH analysis of the *gata1* expression at 22 hpf in WT and *tead1a*-knockdown embryos. (B, C) WISH analysis of the *hbae3* at 48 hpf and o-dianisidine staining at 72 hpf in WT and *tead1a*-knockdown groups. (D-F) QPCR analysis of the phenotype of tead1a MO subgroup in zebrafish tail cut models. At the 72 hpf stage, 12 hours after tail-cut, compared to the control group, the expression of granulocyte markers *cebpα* and *lyz* in the MO group zebrafish larvae significantly decreased (D), the expression of neutrophil-related inflammatory cytokines *il-1β* and *il-6* is significantly decreased (E), while the expression of macrophage markers *mfap4* and *mpeg1* showed no significant difference (F). TC: tail-cut. Error bars represent the mean ± standard deviation (SD) of at least 15-40 embryos or larvae from three independent experiments. Statistical significance was calculated using the Student’s t-test. *, *p* <0.05; **, *p* <0.01; ***, *p* < 0.001; NS, not significant.

Supplemental figure 2. Flow cytometry analysis was performed to quantify the numbers of neutrophils of zebrafish larvae at 72 hpf using the line of Tg(*mpx*:eGFP). (A) Flow cytometry analysis detected 75 out of 10000 cells in the total zebrafish larvae from the line Tg(*mpx*:eGFP) at 72 hpf. (B) Flow cytometry analysis detected 18 out of 10000 cells in the total zebrafish larvae from the line *tead1a^−/−^*//Tg(*mpx*:eGFP) at 72 hpf. Subgroups 1-5 are from WT group, Subgroups 6-10 are from Tg(*mpx*:eGFP) group, Subgroups 11-15 are from *tead1a^−/−^*//Tg(*mpx*:eGFP) group. (C) Gross detection of sorted eGFP positive cells, left for Tg(*mpx*:eGFP) and right for *tead1a^−/−^*//Tg(*mpx*:eGFP). (D) Summary and statistics of flow cytometry detection results of Tg(*mpx*:eGFP) and *tead1a^−/−^*//Tg(*mpx*:eGFP). *P*-value for within-group differences＞0.4, *p*-value for between-group differences < 0.001. Error bars represent the mean ± standard deviation (SD) of at least 100 larvae per subgroup and five subgroups per group and three independent experiments.

Supplemental figure 3. (A) Cellular subpopulation distribution in whole-embryo single-cell sequencing of 3 dpf zebrafish embryos treated with verteporfin. (B) None of the luciferase expressions changed significantly when co-transfected *pu.1* promoter with either *tead1a* or NICD sequence into HEK293T cells. *P*-value for within-group differences＞0.4, *p*-value for between-group differences＞0.05. Error bars represent the mean ± standard deviation (SD) of at least three independent experiments.
